# Peridomestic Infection as a Determining Factor of Dengue Transmission

**DOI:** 10.1371/journal.pntd.0004296

**Published:** 2015-12-15

**Authors:** Ruth Aralí Martínez-Vega, Rogelio Danis-Lozano, Fredi Alexander Díaz-Quijano, Jorge Velasco-Hernández, René Santos-Luna, Susana Román-Pérez, Pablo Kuri-Morales, José Ramos-Castañeda

**Affiliations:** 1 Escuela de Medicina, Universidad de Santander, Bucaramanga, Santander, Colombia; 2 Centro de Investigaciones sobre Enfermedades Infecciosas, Instituto Nacional de Salud Pública, Cuernavaca, Morelos, México; 3 Organización Latinoamericana para el Fomento de la Investigación en Salud, Bucaramanga, Santander, Colombia; 4 Departamento de Control de Vectores, Instituto Nacional de Salud Pública, Tapachula, Chiapas, México; 5 Departamento de Epidemiologia, Universidade de São Paulo. São Paulo, São Paulo, Brazil; 6 Universidad Nacional Autónoma de Mexico-Juriquilla, Santiago de Querétaro, Querétaro, México; 7 Subdirección de Geografía Médica y Sistemas, Instituto Nacional de Salud Pública, Cuernavaca, Morelos, México; 8 Facultad de Medicina, Universidad Nacional Autónoma de México, México D.F., México; 9 Center for Tropical Diseases, University of Texas-Medical Branch, Galveston, Texas, United States of America; Imperial College London, UNITED KINGDOM

## Abstract

**Background:**

The study of endemic dengue transmission is essential for proposing alternatives to impact its burden. The traditional paradigm establishes that transmission starts around cases, but there are few studies that determine the risk.

**Methods:**

To assess the association between the peridomestic dengue infection and the exposure to a dengue index case (IC), a cohort was carried out in two Mexican endemic communities. People cohabitating with IC or living within a 50-meter radius (exposed cohort) and subjects of areas with no ICs in a 200-meter radius (unexposed cohort) were included.

**Results:**

Exposure was associated with DENV infection in cohabitants (PRa 3.55; 95%CI 2.37–5.31) or neighbors (PRa 1.82; 95%CI 1.29–2.58). Age, location, toilets with no direct water discharge, families with children younger than 5 and the House Index, were associated with infection. Families with older than 13 were associated with a decreased frequency. After a month since the IC fever onset, the infection incidence was not influenced by exposure to an IC or vector density; it was influenced by the local seasonal behavior of dengue and the age. Additionally, we found asymptomatic infections accounted for 60% and a greater age was a protective factor for the presence of symptoms (RR 0.98; 95%CI 0.97–0.99).

**Conclusion:**

The evidence suggests that dengue endemic transmission in these locations is initially peridomestic, around an infected subject who may be asymptomatic due to demographic structure and endemicity, and it is influenced by other characteristics of the individual, the neighborhood and the location. Once the transmission chain has been established, dengue spreads in the community probably by the adults who, despite being the group with lower infection frequency, mostly suffer asymptomatic infections and have higher mobility. This scenario complicates the opportunity and the effectiveness of control programs and highlights the need to apply multiple measures for dengue control.

## Author Summary

The study of dengue transmission is essential for proposing alternatives to diminish the cases and the cost of dengue treatment and control. The traditional paradigm establishes that transmission chain starts around a case, but there are few studies that determine the risk, therefore, we studied if to live around a dengue case increases the risk to get infected by Dengue virus. We interviewed and took blood samples from people cohabitating with dengue cases and neighbors in two Mexican communities, to compare we interviewed and took blood samples from subjects of areas without dengue cases in these communities. We found that people cohabitating and neighbors had more risk to get infected. Younger and older person, the workers, families with children younger than 5, houses with toilets with no direct water discharge, and areas with more mosquitoes, also had increased infection risk until one month after the fever onset of dengue case. After this month the frequency of dengue infections was only influenced by the seasonal behavior of dengue and the age of the subjects. Also, we found that 60% of infections are asymptomatic and older people have less risk to develop symptoms. This study suggests that dengue transmission in these locations is initially peridomestic, around the houses of infected subject who may be asymptomatic (without symptoms), and it is influenced by other characteristics of the individual, the neighborhood and the community. After this peridomestic transmission, dengue spreads in the community probably by adults who mostly suffer asymptomatic infections and have higher mobility, which complicates the application and affects the results of vector control programs.

## Introduction

Dengue is the most important vector-borne viral disease in the world due to its morbidity and economic impact [1]. Because there are no vaccines available to date, the interruption of dengue transmission is based on the vector control. However, despite control programs in endemic countries, the persistence and dispersion of the disease imply a review of the epidemiologic assumptions in which vector control is based on.

It is generally assumed that dengue transmission mainly depends on vector density and although there is an association between dengue incidence and the increase of entomological indices at the regional level, in endemic locations this association is controversial [2,3,4]. In Mexico, the perifocal control, which consists on modifying environments around the home of dengue cases, is the main component of the vector control program and has not proved to decrease dengue incidence. This may be due to the unsuitable application and vector resistance to pesticides, lack of continuity of control measures or because transmission is not occurring around cases or occurring at a lower rate than expected. The evidence of peridomestic transmission is also controversial; in Thailand, there were more Dengue virus (DENV) infections reported in cohabitants and neighbors of dengue cases compared to neighbors of febrile subjects without dengue, but in Nicaragua, the infection frequency was similar between these groups [5–7].

In addition, perifocal control is based on cases reported to National System of Epidemiologic Surveillance (SINAVE). However, the asymptomatic infections are around to 50% [8–19] and at least 25% of them present viremia [6,11,12,20,21]. Therefore, their contribution to transmission may be considerable for maintaining endemicity and may influence the impact of any vector control measure. Other subject characteristics such as age, intra-locality mobility, some housing characteristics and population density, have also been associated with increased infections [2,22–25].

Identifying factors associated with DENV infection and clarifying dengue transmission pattern in endemic areas would allow to improve prevention and control programs in order to have a greater impact on the dengue burden. In this work we test the hypothesis of dengue transmission occurring mainly in the peridomestic area of Index Cases (ICs) in two Mexican endemic communities; additionally, the relation of some sociodemographic, environmental and vector density variables was determined

## Materials and Methods

### Design and study population

We carried out a prospective cohort study in 5 years old (y/o) or older living in Axochiapan and Tepalcingo, Morelos, Mexico. Subjects not susceptible to follow-up were excluded. The study protocol was previously published [26]. The calculated sample size was 1,178 subjects. A summary of the used methods is presented below, mentioning the changes in the protocol.

### Recruitment and Follow-up

The exposed cohort included subjects living in the residence of ICs or even in up to 4 neighbor houses within a 50-meter radius. The unexposed cohort included subjects living in areas where no ICs were reported within a 100-meter radius in the 2 months previous to the sampling day; from these areas, up to 5 houses were included in 50-meter radius around the first house where at least one subject accepted to participate. Per each group exposed to an IC (exposed group) an unexposed group was enrolled in the same location during the following three weeks (Tepalcingo, median: 6 day, range: 0–8; Axochiapan, 17.5 days, 2–22 days).

Two visits were carried out, baseline between June and November 2011, and follow-up between August 2011 to March 2012. In each visit, an interview that included the presence of symptoms was performed, and a blood sample was taken in order to measure DENV antibodies, as describe in the protocol [26], but the follow-up visit was made between week 12 and 18 and the active telephonic surveillance was carried out at least once a month. Passive surveillance was also carried out consulting the “Unique Automated System for Epidemiologic Surveillance (SUAVE)”. Variables such as working/studying outside the locality, leaving the locality in the past 15 days and hours of the day staying home in a working day or holiday, and occupation were evaluated. Previous history of DENV exposure (seroprevalence), determined in basal serum by indirect IgG ELISA (Panbio, E-DEN-01G) following manufacturer recommendations, was also considered.

Regarding the vector, each house was geolocated using GPS; a questionnaire was applied and the yard and water containers were inspected at each visit. Home and neighborhood control actions carried out by subjects, the Health Services of Morelos (SSM) or the municipality, and mosquito relative abundance were assessed. At the housing level, larva/pupae home-infestations and Container Index were considered. In each group of neighbor houses within a 50-meter radius, the Breteau Index and the House Index, were determined. Environment and weather variables were also measured.

### Definition of recent DENV infection

A recent infection by DENV, symptomatic or asymptomatic, was considered whenever IgM or IgG capture ELISA tests resulted positive in any blood sample (Panbio Cat No. E-DEN01M y E- DEN02G). The tests were carried out and interpreted according to manufacturer recommendations. Furthermore, infections were considered recent when suspected dengue case were confirmed by the SSM [26].

Recent infection was subclassified as 1. Pre-enrollment infection when IgM or IgG capture ELISA was positive in the baseline sample or when a subject was confirmed dengue by the SSM. A subject was considered with no pre-enrollment infection when the two capture tests in the baseline sample were negative. We decided to include positive IgG capture subjects as recent infections, because the people who live in these towns show high seroprevalence (>80% in 25 and older), and it has been observed that in secondary infections negative IgM subjects could still develop an IgG response [27, 28]. 2. Post-enrollment Infection when IgM and IgG capture tests were negative in the baseline sample and any test was positive in the following-up sample or the case was confirmed by the SSM (S1 Table). A subject was considered as having no post-enrollment infection when the two capture tests were negative in the baseline and follow-up samples. Otherwise, a symptomatic infection was considered when the subject presented fever in the two months before the baseline evaluation in pre-enrollment infection or when the subject presented at least a fever once at some point during follow-up in post-enrollment infections.

### Statistical Analysis

**Fig 1:**
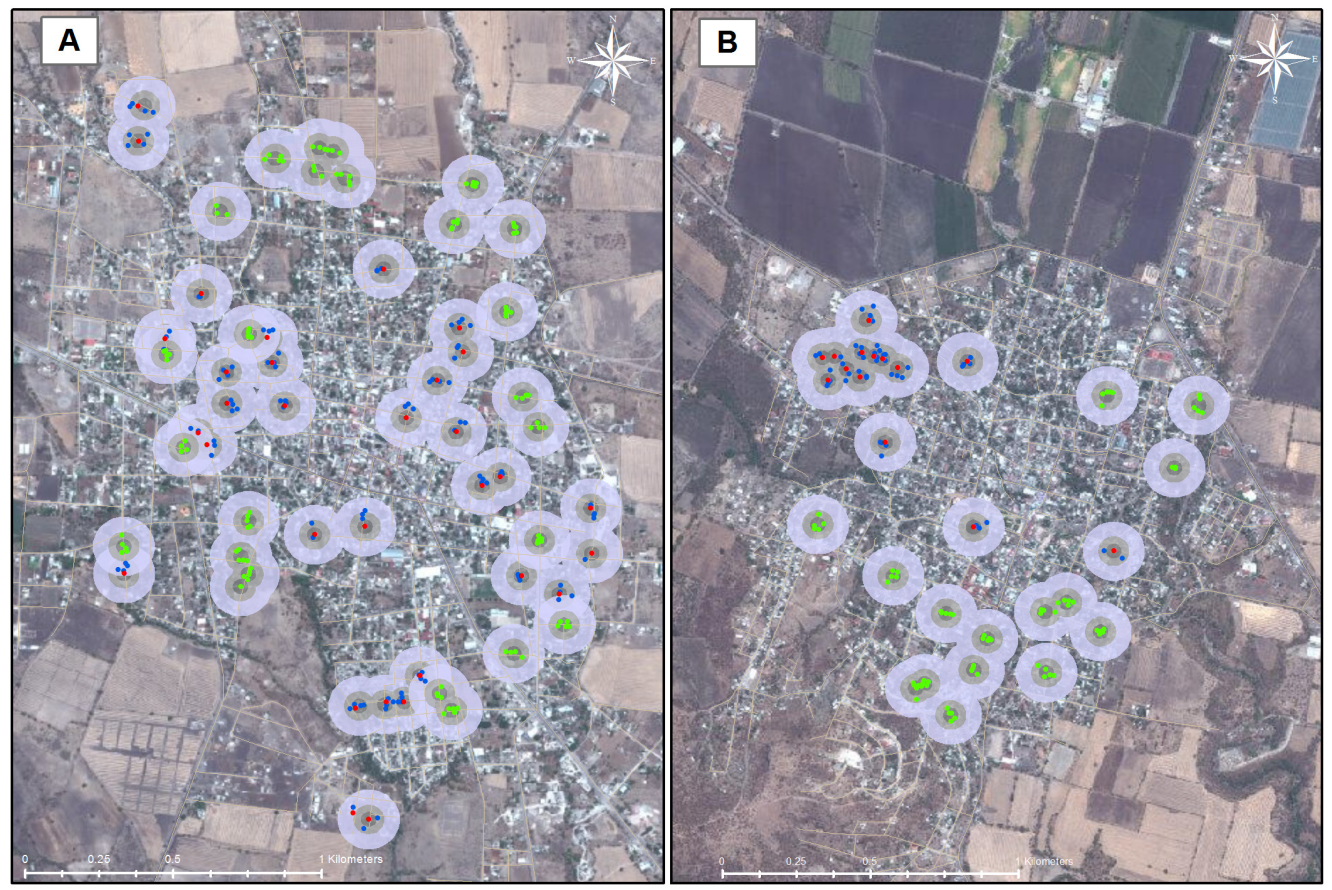
Accumulated Distribution of Groups in Axochiapan (A) and Tepalcingo (B). IC houses in red, IC neighbor houses in blue (exposed group) and unexposed group houses in green. Areas have 50, 100 and 200 meters in diameter with the center at the IC house or in the centroid of unexposed the neighbor houses. Source: Esri, DigitalGlobe, GeoEye, i-cubed, USDA, USGS, AEX, Getmapping, Aerogrid, IGN, IGP, swisstopo, and the GIS User Community.

Initially, house locations from Global Positioning System were verified using satellite photography; ICs reported in the SINAVE [29] and not evaluated in the cohort, were located on a map. To verify the house groups and the peridomestic exposure in the area, buffer areas of 200 m, 100 m, and 50 m in diameter were traced, with the center at the IC house, using ArcGIS 10 software. Considering the 200 m buffer, ICs symptoms starting date and unexposed group recruitment date, 5 unexposed groups had to be reclassified as exposed in Axochiapan (Fig 1). The Fig 1 was developed in ArcGIS 10 Software of ESRI, the ArcGIS Software include access to Map Image Services web property of ESRI. The map Image Services includes NASA Blue Marble: Next Generation 500m resolution imagery at small scales (above 1:1,000,000), i-cubed 15m eSAT imagery at medium-to-large scales (down to 1:70,000) for the world, and USGS 15m Landsat imagery for Antarctica. The map also includes i-cubed Nationwide Prime 1m or better resolution imagery for the contiguous United States, Getmapping 1m imagery for Great Britain, and GeoEye IKONOS 1m resolution imagery for Hawaii, parts of Alaska, and several hundred metropolitan areas around the world. I-cubed Nationwide Prime is a seamless, color mosaic of various commercial and government imagery sources, including Aerials Express 0.3 to 0.6m resolution imagery for metropolitan areas and the best available United States Department of Agriculture (USDA) National Agriculture Imagery Program (NAIP) imagery and enhanced versions of United States Geological Survey (USGS) Digital Ortho Quarter Quad (DOQQ) imagery for other areas. For more information on this map, visit us online at http://goto.arcgisonline.com/maps/World_Imagery

Comparisons were made for both individual and housing models, using the Chi-squared or Mann-Whitney tests. The proportions (pre-recruitment infection prevalence and post-recruitment infection incidence) were analyzed using binomial regression. When there were convergence problems the adjusted measures of association were obtained using Breslow-Cox regression with a constant in the time variable and the robust option [30–32]. Counting of pre-recruitment infections was modeled using Poisson regression. According to the analysis level, an adjustment of standard errors was made considering the 359 houses as cluster units for individual analysis; and, the 91 clusters (houses in 50 meters around IC) for comparison of housing. In such models, only variables based on subject matter knowledge and p<0.20 value were evaluated by manual backward elimination, keeping those variables with a p<0.05 or those that modified the estimator of IC exposure by more than 10%. Subsequently, eliminated variables during the backward elimination process were evaluated in the resulting model, one by one. Additionally, we tested vector indexes, month of recruitment and family composition. After, this resulting model was evaluated with a multilevel regression, with two and three levels, with random and mixed effects. We use de Akaike's Information Criterion (AIC) to select the better model. The analysis was made with Stata SE 12.

The study was approved by the Instituto Nacional de Salud Pública ethical commission (CI: 986, No. 1032). Each subject, or one of the parents of the minors, signed the informed consent. Also, written approval was requested for subjects between 7 and 17 years of age.

## Results

### Description of ICs

46 ICs were visited (31 in Axochiapan); 45% were men; there was only one case younger than 5; 28.3% were reported as hemorrhagic dengue fever. 95.7% were visited between 11 and 33 days after the fever onset. In 39.1% of the houses, inhabitants had carried out some kind of measure against the vector within the last month, contrasting with those carried out by the SSM (91.3%) or by the local government (69.6%); 15.2% of the houses were infested with *Aedes* larvae/pupae. The serotype from IC was DENV-1.

### Cohort Characteristics

**Fig 2:**
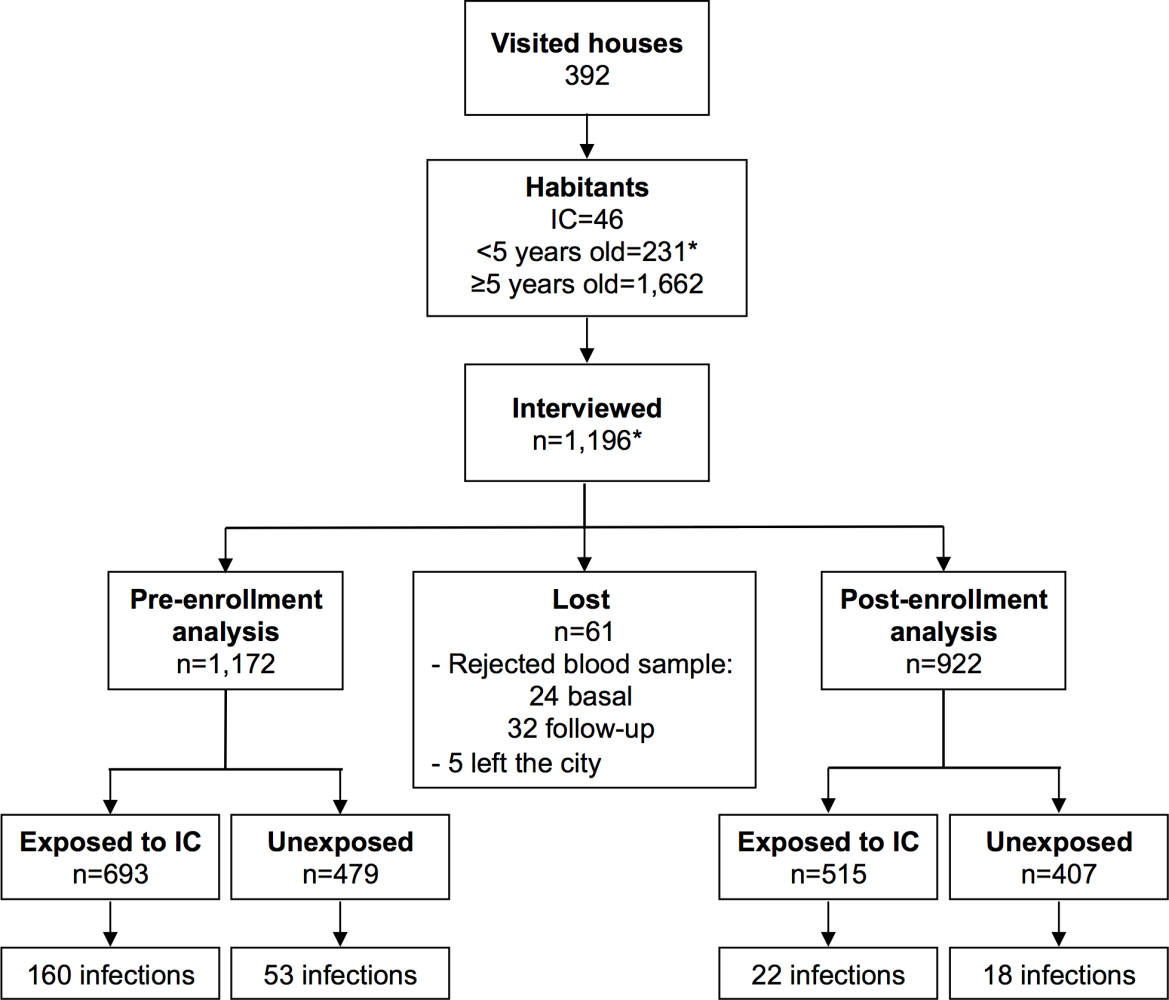
Cohort Enrollment and Follow-up. *Two 3-year-old children were included because they had pre-enrollment symptomatic infections diagnosed by the SSM.

46 exposed groups (15 in Tepalcingo) from the ICs and 44 unexposed groups (15 in Tepalcingo) were selected. 392 houses were visited, 1,893 subjects lived in them, in addition to the ICs. 231 of them were younger than 5; however, 2 of them were included in the cohort because they were pre-enrollment infection diagnosed by the SSM. Out of the 1,662 subjects older than 5, 1,196 were interviewed (71.8%) (Fig 2). No significant differences were present among participants and non-participants (468, 28.2%) regarding location (p = 0.26) and age (p = 0.31), but there were differences for gender (68.7% non-participating men vs. 41.7% of participants, p<0.001). The losses were not differential between exposed and unexposed (5.2% vs 5%, p = 0.854).

After the reclassification of 5 groups, 58.4% of the houses were exposed and 65.6% were located in Axochiapan. The exposition was differential between localities (p = 0.001). In exposed houses a greater frequency of adulticide application due to focal fumigation around ICs was observed. House Index and Breteau Index showed a significant difference (Table 1).

**Table 1:** Description of Houses from the Cohort.

Characteristic	Total (n = 392)	Unexposed (n = 163)	Exposed (n = 229)	p
**Cohabitants** Median (range)	5 (1–21)	5 (1–15)	5 (1–21)	0.834
**Children under 5 years of age** n (%)	163 (41.6)	76 (46.6)	87 (38)	0.087
**Location**				**0.001**
Tepalcingo	135 (34.4)	71 (43.6)	64 (28)	
Axochiapan	257 (65.6)	92 (56.4)	165 (72.1)	
**Toilet**				0.111
Direct discharge	206 (52.6)	80 (49.1)	126 (55)	
Manual discharge	174(44.4)	78 (47.9)	96 (41.9)	
Toilet with no water intake	4(1.0)	0	4 (1.8)	
No toilet available	7 (1.8)	5 (3.1)	2 (0.9)^a^	
**Piped water availability**				0.576
Public network within the house	300 (76.5)	130 (79.8)	170 (74.2)	
Public network outside the house but within housing grounds	10 (2.6)	3 (1.8)	7 (3.1)	
Water from a public faucet or hydrant	3 (0.8)	0	3 (1.3)	
Water from another house	3 (0.8)	1 (0.6)	2 (0.9)	
Water from a water tank truck	4 (1.0)	1 (0.6)	3 (1.3)	
Water from a well	72 (18.4)	28 (17.2)	44 (19.2)	
**Socioeconomic level** n (%)				0.211
Low	131 (33.5)	55 (33.7)	76 (33.3)	
Medium	130 (33.3)	61 (37.4)	69 (30.3)	
High	130 (33.3)	47 (28.8)	83 (36.4)	
**Mosquito nets in windows**				0.522
None	215 (54.9)	86 (52.8)	129 (56.3)	
All of them	93 (23.7)	38 (23.3)	55 (24)	
Some of them	83 (21.2)	38 (23.3)	45 (19.7)	
**Measures against the vector within the last month**				
**Carried out by the subjects**	198 (50.5)	83 (50.9)	115 (50.2)	0.697
House fumigation	114 (29.1)	45 (27.6)	69 (30.1)	0.409
Breeding elimination	62 (15.8)	26 (16)	36 (15.7)	0.489
Other measures^b^	81 (20.7)	34 (20.9)	47 (20.5)	0.489
**Carried out by the SSMs**	277 (70.7)	89 (54.6)	188 (82.1)	**<0.001**
Larvicides	93 (33.6)	66 (74.2)	27 (14.4)	**<0.001**
Adulticides	95 (34.3)	14 (15.7)	81 (43.1)	
Larvicides and adulticides	89 (32.1)	9 (10.1)	80 (42.6)	
**Carried out by the Municipality**	239 (61)	96 (58.9)	143 (62.5)	0.283
Larvicides	8 (3.4)	3 (3.1)	5 (3.5)	0.117
Adulticies	198 (82.9)	73 (76)	125 (87.4)	
Larvicides and adulticides	16 (6.7)	9 (9.4)	7 (4.9)	
Dejunking	13 (5.4)	9 (9.4)	4 (2.8)	
Other combinations of actions	4 (1.7)	2 (2.1)	2 (1.4)	
**Infested Houses (larvae/pupae)**	88 (22.5)	44 (27)	44 (19.2)	0.069
**Containers Index** ^c^ **Median (IQR)**	0 (0–0%)	0 (0–5.3%)	0 (0–0%)	0.111
**House Index** ^d^	20 (0–40)	20 (0–40)	20 (0–33.3)	0.0001
**Breteau Index** ^d^	20 (0–50)	20 (20–60)	20 (0–40)	0.0001

^a^An exposed house did not specify (0.4%) the type of toilet.

^b^Fishes, fumes, mosquito coils, private application of abate, repellent, bed nets.

^c^Containers Index = (# of infested containers/ # of inspected containers with water)x100.

^d^Obtained for each group of neighbor houses within a 50-meter radius. House Index = ([# of positive houses / # inspected houses in the group]x100). Breteau Index = ([# of positive containers / # of inspected houses in the group]*100).

IQR: Interquartile Range.

59.5% (712/1,196) of the subjects were exposed. Among the exposed and the unexposed, a significant difference was observed in the health insurance (p = 0.001), the location where the subject lives (p <0.001), fever reports within the last two months and nutritional status (p <0.001) (Table 2). Seroprevalence was 43.5% in subjects 5 to 14 y/o and higher than 92% in older than 35; both globally and per decade, seroprevalence was similar between exposed and unexposed subjects.

**Table 2:** Description of Participating from the Cohort.

Characteristic	Total (n = 1,196)	Unexposed (n = 484)	Exposed (n = 712)	p
**Age** Median (range)	30 (3–86)	31 (5–85)	29 (3–86)	0.213
**Sex** Male n (%)	499 (41.7)	197 (40.7)	302 (42.4)	0.555
**Occupation**				0.094
Student	296 (24.8)	111 (22.9)	185 (26)	
Student and Employee	37 (3.1)	10 (2.1)	27 (3.8)	
Employee	224 (18.7)	84 (17.4)	140 (19.7)	
Independent	211 (17.6)	84 (17.4)	127 (17.8)	
Housewife	345 (28.9)	156 (32.2)	189 (26.5)	
Other	83 (6.9)	39 (8.1)	44 (6.2)	
**Education in older than 6**	**n = 1152**	**n = 472**	**n = 680**	0.084
Illiterate	123 (10.7)	62 (13.1)	61 (9)	
Reads and Writes	309 (26.8)	122 (25.9)	187 (27.5)	
Elemental or Middle School	550 (47.7)	227 (48.1)	323 (47.5)	
High School or more	170 (14.8)	61 (12.9)	109 (16)	
**Insurance**				**0.001**
Seguro Popular	860 (71.9)	378 (78.1)	482 (67.7)	
Uninsured	185 (15.5)	66 (13.6)	119 (16.7)	
IMSS: Instituto Mexicano del Seguro Social	71 (5.9)	17 (3.5)	54 (7.6)	
ISSSTE: Instituto de Seguridad y Servicios Sociales de los Trabajadores del Estado.	68 (5.7)	20 (4.1)	48 (6.7)	
**Location**				**<0.001**
Tepalcingo	392 (32.8)	216 (44.6)	176 (24.7)	
Axochiapan	804 (67.2)	268 (55.4)	536 (75.3)	
**Has always lived in Morelos**	960 (80.3)	395 (81.6)	565 (79.4)	0.336
**Fever in the last 2 months**	201 (16.8)	52 (10.7)	149 (20.9)	**<0.001**
Consultation due to fever	110 (54.7)	27 (51.9)	83 (55.7)	0.637
Private consultation	29 (26.6)	9 (33.3)	20 (24.1)	0.344
**Nutritional State**				**0.026**
Malnutrition	26 (2.4)	11 (2.4)	15 (2.3)	
Normal	431 (39)	186 (41.3)	245 (37.4)	
Overweight	336 (30.4)	114 (25.3)	222 (33.8)	
Obesity	313 (28.3)	139 (30.9)	174 (26.5)	
**Socioeconomic Level**				**0.007**
Low	399 (33.5)	157 (32.4)	242 (34.1)	
Medium	402 (33.7)	187 (38.6)	215 (30.3)	
High	392 (32.9)	140 (28.9)	252 (35.5)	
**Losses**	61 (5.1)	24 (5)	37 (5.2)	0.854
**Seroprevalence** (positive baseline indirect IgG)	887/1162 (76.3)	362/479 (75.6)	525/683 (76.9)	0.858

### Recent DENV Infections in the Cohort

253 recent infections were identified, 99 subjects (39.1%) reported fever sometime during the two months prior to recruitment (pre-enrollment infections) or during follow-up (post-enrollment infections). Out of these, 33 did not seek medical attention (33.3% underreported); and out of those 66 subjects who consulted a doctor, 45 were not reported to the SINAVE (68.2% undernotified). Importantly, no one of the 17 dengue infections that consulted to the private sector were reported to the SINAVE.

**Fig 3:**
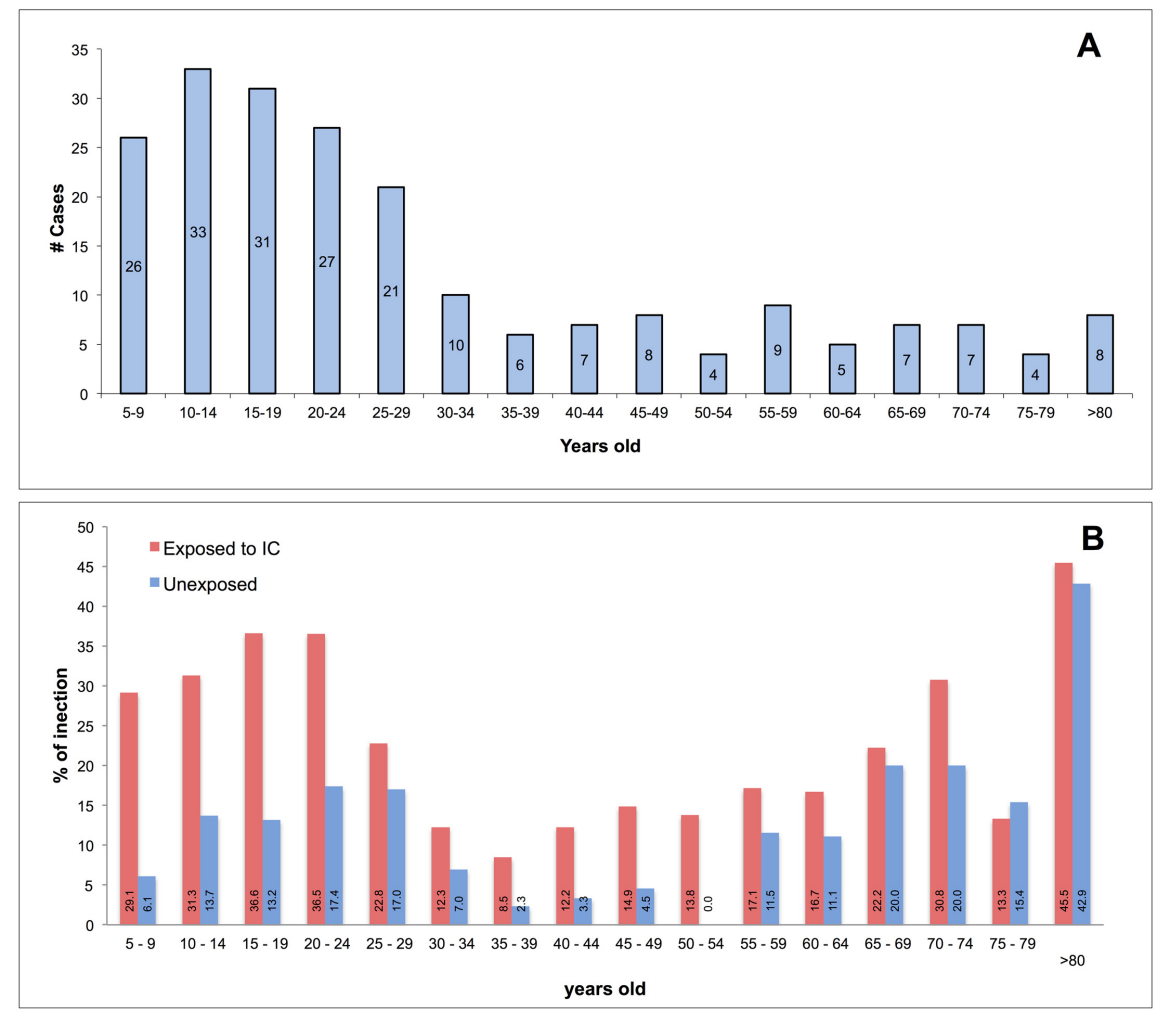
Pre-enrollment DENV Infections per Age. A. Pre-enrollment infection cases (n = 213/1,172), B. Frequency of pre-enrollment infections.

**Fig 4:**
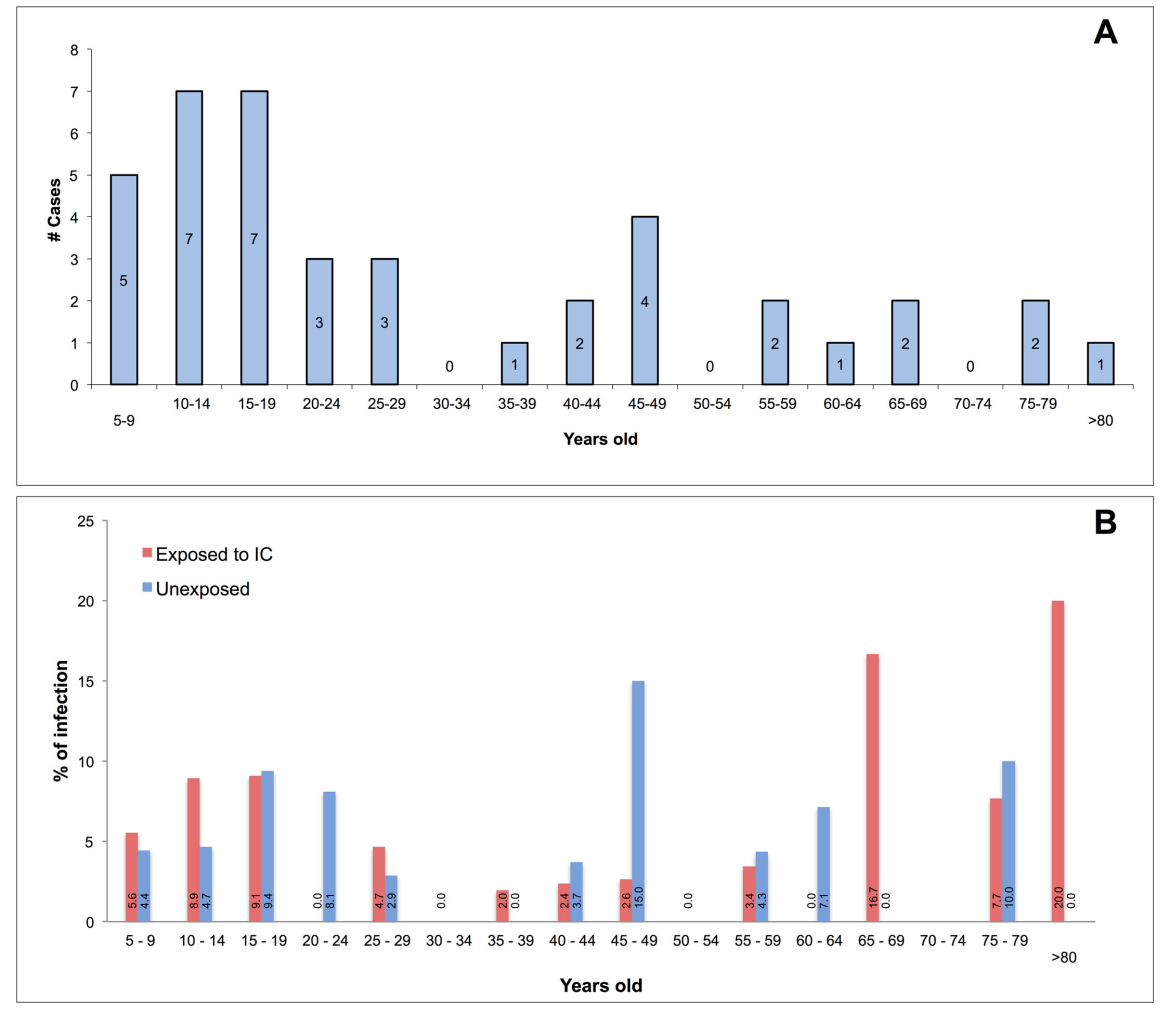
Post-enrollment DENV Infections per Age. A. Post-enrollment infection cases (n = 40/922), B. Frequency of post-enrollment infections.

60.9% of infections were asymptomatic. A relation between asymptomatic infection and age was observed; younger presented a lower percentage of asymptomatic infections (p<0.001). The median age for asymptomatic infections was 27 y/o (Interquartile Range—IQR 16–54) and for symptomatic infections was 16 y/o (IQR 10–25) (Mann-Whitney, p<0.0001). A greater age was a protective factor for the presence of symptoms (Risk Ratio—RR 0.98; 95%CI 0.97–0.99). Even though recent infections were detected in the all age groups, most were present in those younger than 30 (Figs 3A and 4A). Recent infection was 27% (182/675) in exposed subjects and 15.4% (71/460) in unexposed subjects (RR 1.73; 95%CI 1.37–2.19). 213 (84.2%) were pre-enrollment and 40 (15.8%) post-enrollment infections.

### Pre-enrollment DENV Infections

Present in all age groups, but a greater infection frequency was observed in younger than 30 and older than 64 (Fig 3A and 3B). Out of 392 visited houses, subjects were evaluated in 388 of them, 62.1% (241) did not present any infection, 25.8% (100) presented at least one pre-enrollment infection, 8.8% (34) presented two, 2.3% (9) presented three, 0.5% (2) presented four and 0.5% (2) presented up to five infections. The Prevalence Ratio to IC exposure was 1.96 (95%CI 1.5–2.56). An association was also observed with locality (Axochiapan), age, and the presence of younger than 5 in the houses, but weather variables were not associated. In multivariate analyses, consistency among the used methods for found relationships was observed (Table 3, S2 Table). An increased risk was observed with proximity to an IC, locality and younger than 30 and older than 64. The occupation (workers) and the insurance (government health coverage), that were associated in the Breslow-Cox Regression, lost the association in the multilevel analysis (p = 0.059 and p = 0.053, respectively), but were remained in the model because the AIC of this model was lower (Table 3).

**Table 3:** Multilevel Pre-enrollment Infection Models (n = 1,172 subjects; 213 infections).

Variable	n	Infectedn (%)	PR	Model 1 (with individual-level variables) PRa (95% CI)	Model 2 (with contextual-level variables) PRa (95% CI)
**Main exposition**					
**Exposure to an Index Case (IC)**					
Unexposed	479	53 (11.1)	1		1
Lives within 50m of an IC	534	107 (20)	**1.81 (1.33–2.46)**		**1.82 (1.29–2.58)**
Cohabits with an IC	159	53 (33.3)	**3.01 (2.15–4.21)**		**3.55 (2.37–5.31)**
**Individual variables**					
**Age**					
< 30 years old	573	138 (24.1)	**2.42 (1.79–3.27)**	**2.8 (1.94–4.05)**	**2.94 (2–4.34)**
30–64 years old	492	49 (10)	1	1	1
> 64 years old	107	26 (24.3)	**2.44 (1.59–3.74)**	**2.5 (1.53–4.08)**	**2.68 (1.64–4.40)**
**Occupation**					
Student	320	66 (20.6)	1	1	1
Non-studying workers	426	74 (17.4)	0.84 (0.62–0.14)	1.39 (0.95–2.02)	1.45 (0.99–2.14)
Housewives and others^a^	426	73 (17.1)	0.83 (0.62–1.12)	1.22 (0.84–1.79)	1.33 (0.90–1.96)
**Insurance** ^b^					
Uninsured	183	45 (24.6)	1	1	1
Seguro Popular	840	139 (16.5)	**0.67 (0.50–0.90)**	0.75 (0.53–1.06)	0.71 (0.50–1)
Other	145	29 (20)	0.81 (0.54–1.23)	0.97 (0.6–1.57)	1.01 (0.62–1.65)
**Contextual variables**					
**Location**					
Tepalcingo	386	49 (12.7)	1		1
Axochiapan	786	164 (20.9)	**1.64 (1.22–2.21)**		**1.65 (1.18–2.31)**
**Toilet**					
Direct discharge	573	97 (16.9)	1		1
Other	599	116 (19.4)	1.14 (0.90–1.46)		**1.38 (1.04–1.83)**
**Younger than 5 at home** ^c^					
No	638	100 (15.7)	1		1
Yes	534	113 (21.2)	**1.35 (1.06–1.72)**		**1.34 (1–1.8)**
**No. of cohabitants between 13 and 29 years old** ^d^		2 (1–2)	1.04 (0.96–1.13)		**0.88 (0.79–0.98)**
**House Index (10 points %)** ^d^		20 (0–60)	1.04 (0.98–1.10)		**1.1 (1.03–1.17)**
**Log-likelihood value**				-554.56	-526.56
**AIC**				1125.13	1083.13

^a^Others including unemployed, retirees, handicapped persons.

^b^Insured status not specified in 4 uninfected subjects.

^c^Family members were categorized: <5 y/o, between 5 and 12, between 13 and 29, between 30 and 64, and older than 64.

^d^Median (Interquartile Range).

PR: Prevalence Ratio. PRa: Adjusted Prevalence Ratio.

Regarding family structure, the presence of younger than 5 was associated with a higher risk of infection, both in the individual and the house model. On the other hand, in the individual model, the number of cohabitants between 13 and 29 y/o was associated with protection, but in the house model, protection was associated with the 30 to 64 y/o group. Also in individual model, having a toilet with no direct discharge was associated with a higher infection risk. Both in individual and house models, a higher risk was related to vector density, as evaluated with Breteau Index and House Index. The final model included the House Index, as both indexes presented a similar explanation and this one was easier to measure. We observed that with every 10 percent increase in House Index, pre-enrollment infection increased by 10% (Table 3, S2 Table).

### Post-enrollment DENV Infections

During follow-up, new infections occurred in almost all age groups (Fig 4A and 4B). Of 359 houses followed, 21 (8.7%) presented at least one infection, 3 (0.8%) presented two, and 1 (0.3%) presented three infections during follow-up. Living in the peridomestic area of an IC did not increase the risk of new infections (RR 0.97; 95%CI 0.53–1.78). In the individual multilevel model, an increased risk infection was observed in younger than 30 (Adjusted Risk Ratio—RRa 2.42; 95%CI 1.16–5.04) compared to the 30 to 64 y/o group; although older than 64 exhibed similar tendency, it was not significative (RRa 2.89; 95%CI 0.99–8.47, p = 0.052). Furthermore, the June-August enrollment period was associated with post-enrollment infections (Individual: RRa 3.82; 95%CI 2.05–7.12 and House: 2.56; 95%CI 1.32–4.98) compared to those enrolled between September-November 2012. Likewise, infection risk increased 17% at the house per each additional family member between 5 and 29 y/o (RRa 1.17; 95%CI 1.01–1.36). When the dependent variable was analyzed as a rate using the Poisson model, the use of adulticides in the baseline evaluation was associated with a lower infection rate in houses (Incidence Rate Ratio 0.50; 95%CI 0.26–0.96); however, this relationship losses statistical significance when adjusting for enrollment month.

## Discussion

This is the first study that has estimated the magnitude of the association between a dengue case detected by SINAVE and the occurrence of other peridomestic infections with a follow-up longer than 21 days. The frequency of recent infections around an IC (27%) was similar in Indonesia (24.5%) and in a multicenter study (25.6%), but greater than in Thailand (12.4% and 16%), which may have occurred due to a longer follow-up and the inclusion of older than 15, and in Vietnam (11.9%) because in this study IgM was the only criteria used to define recent infection [5,7,10,21,33]. Our data contrasts with the low infection frequency in Nicaragua (2.4%), where there is probably a lower force of infection [6].

In the current study, we demonstrate that the risk to get infected living around 50 meters of a IC (vicinity), drops to the half comparing with the risk of a cohabitant of a IC, therefore it is highly possible that the risk disappear after a equivalent distance away. Since the estimated flying range of the vector is about 500 meters, and the highest risk is inside the first 50 meters, therefore, the vector is for all that matters static (Table 3). Likewise, in Thailand, a gradient of infection frequency was observed depending on the distance to the IC houses and a higher frequency of DENV infected mosquitoes in IC houses. This findings in small-scale space-time groups support the hypothesis of dengue transmission being predominantly perifocal and that it is likely dengue spreads in a location by the movement of infected humans more than by the movement of infected mosquitoes [7,25,34].

The frequency of recent infections in the unexposed group (15.4%) was greater than the reported in Thailand (0% and 1.1%), Nicaragua (2.5%) and Vietnam (5.1%); this may be explained by the larger follow-up time, the inclusion of older than 15 [5–7,33] or possibly by unexposed group contamination due to suspicious dengue cases that were not diagnosed in Axochiapan (42.5%) and in Tepalcingo (18.7%) or to underreported/undernotified cases (78.8%), especially subjects consulting private clinics, who were not reported to the SINAVE.

Furthermore, age was independently associated with pre-enrollment infection (Fig 3B). Interestingly, the infection rate in older than 64 was similar to younger than 30; this may be because they remain longer where there is infected vector. In line with this interpretation, we found an marginal association with occupation, since, compared to students, workers had a greater infection risk, similar to housewives/other, even though trend were non-significant because of a lack of power. This could occur, because health promotion school campaigns are carried out in these locations; this could make schools places with lower vector density than houses, work places or other destinations. Also, we have the hypothesis that the students, which are younger with higher DENV infection frequency, get infected in their own houses.

Independently living in Axochiapan was also associated with pre-enrollment infection in agreement with the incidence detected by the SINAVE in 2011. Regarding house characteristics, we find a higher risk in those with toilets with no direct discharge, which probably reflects the way water is used and this is related with an increase of potential breedings. We also found an association with the age structure of the family; families had highest risk if they had children younger than 5. We can speculate they need care providers that must spend more time in houses where the transmission is occurring, or these children have a greater frequency of primary infections, which would present higher viremias and consequently they would transmit the infection more efficiently to nearby vectors.

On the other hand, we found asymptomatic infections accounted for 60% of the total, similar to the magnitud determined in Brazil and Thailand [5,13,16,18]. The frequency of asymptomatic infections increases with age which is in contrast with the observation in Nicaragua where symptomatic subjects were on average 1.2 years older than asymptomatic, but the cohorts have demographic differences [35]. Factors related to asymptomatic infection include the time between infections, infection type (post-secondary, primary or secondary) and dengue incidence during the past year, and we found evidence of some of those in our study [35–37]. A significant proportion of asymptomatic subjects shows a detectable viremia [6,8–21]. Furthermore, it was found that at least 75% of hospitalized and outpatient cases exceed the viremia threshold required to infect 50% of *Aedes aegypti*, despite outpatients presenting lower viremia [38]. Overall, these findings suggest that asymptomatic subjects could participate in DENV transmission. Overall, these findings suggest that asymptomatic subjects could participate in DENV transmission. If asymptomatic subjects are relevant in the transmision the consequences of these on surveillance and control programs will be enormous, hence further prospective studies must be conducted to clarify this point.

We observed that risk to IC exposure disappears over time because post-enrollment infection was not associated with this exposure, similar to observed in Vietnam [33]. Also, vector density was not associated, probably because once transmission starts, it is maintained by a basal vector level. However, post-enrollment infections were associated with the age and dengue local seasonal behavior (enrollment period), and family composition (subjects between 5 and 29 y/o).

We observed that peridomestic transmission is the main determinant of endemic incidence and accordingly we found high frequency of perifocal antivectorial activities and low entomological indexes in the exposed groups, however there was a high frequency of pre-enrollment infections (Table 1), which may be interpreted as effectiveness on vector indexes but with low impact on perifocal transmission. This may be explained by delayed of control measures, because when a case is detected by the SINAVE an accumulation of asymptomatics and underreported/undernotified symptomatics may have previously occurred, which may start and maintain dengue transmission, or the infection is occurring almost simultaneously in the ICs, cohabitants and neighbors. Although, there are a report from Cuba in which the risk to be infected is associated with high House Index and Breteau Index at the vecinity of ICs [4], in general, there is no evidence that perifocal control impacts dengue transmission, in part because the sensitivity of the SINAVE, but also because the participation of asymptomatic subjects [39]. However, considering that had association between pre-enrollment infections and vector density, we propose to strengthen vector control activities that are carried out throughout the year and to conduct specific studies to evaluate the impact of these activities and perifocal activities on local DENV transmission.

The main limitation of this study is that the start of the transmission chain could not be established because pre-enrollment infections may have occurred one or two months before IC. This was because subjects were enrolled to one a month after the IC fever onset, serologic tests can remain positive during this time and most infections were asymptomatic; even then, we could establish a peridomestic risk pattern that will allow to conduct more specific studies to determine how the transmission chain begins.

**Fig 5:**
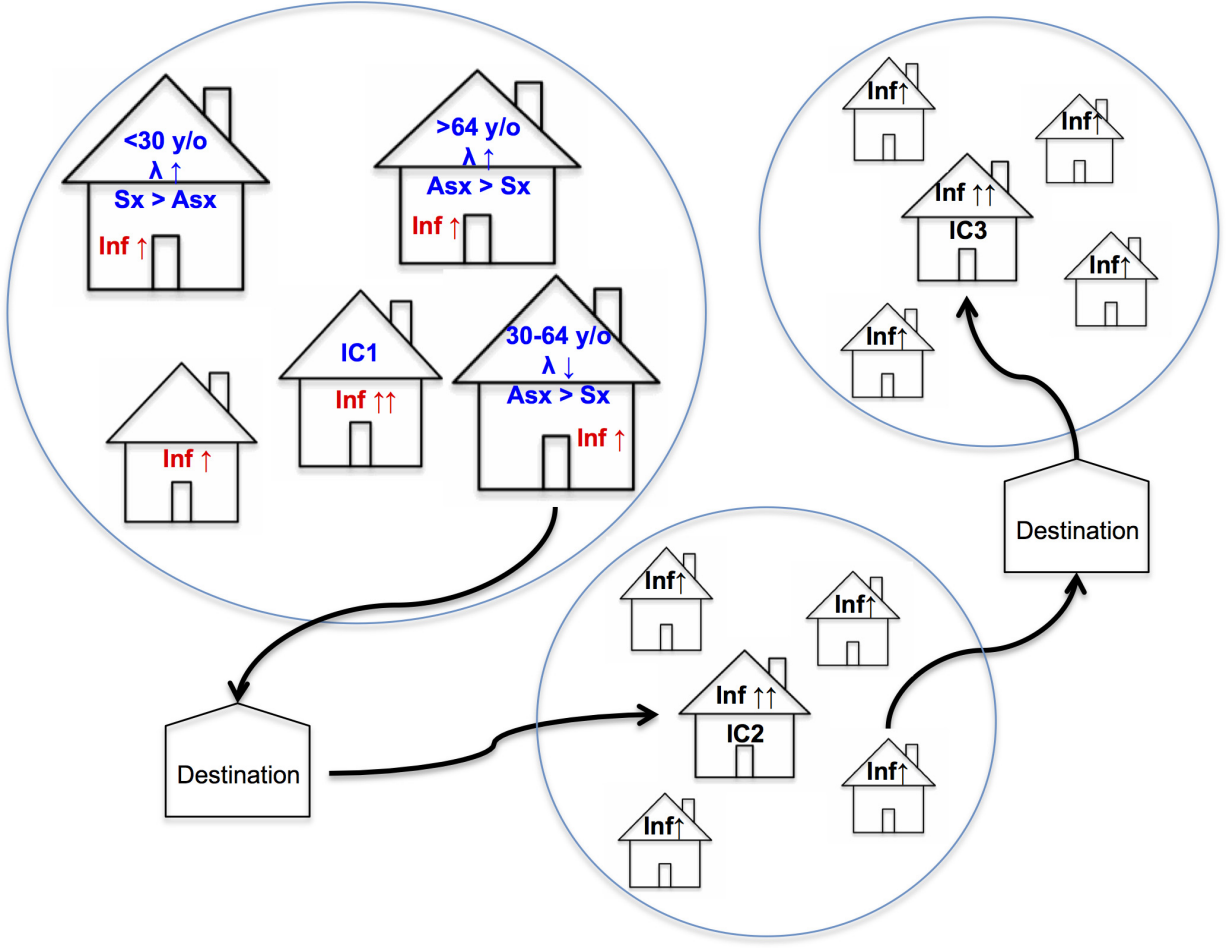
Proposed Dengue Transmission in Studied Communities. Force of infection (λ). Symptomatic (Sx). Asymptomatic (Asx). Index Case (IC). Infection (Inf). Years old (y/o). Destination, includes workplace and other locations where subjects spend enough time to be bitten by vectors located in that area; except for schools.

Taking together all the data, we propose the hypothesis that in these communities, dengue endemic transmission is initially peridomestic for about 3 months, since we detected high recent infection frequency in exposed groups (Table 3); after 3 months the risk became equal in both exposed groups and unexposed groups (RR 0.97; 95%CI 0.53–1.78). As a consequence, the dengue peak during the year is determined by this period of peridomestic transmission. Also, the decrease of infection incidence related to the increase of distance from IC house occurs because the vector is essentially static (Table 3). Consequently, the spread of infection within a community will mainly depend on human mobility (Fig 5). In this sense, we propose subjects between 30 and 64 years old, despite they probably have lower force of infection, being asymptomatic and economically active, move to daily destinations where they remain long enough to be bitten by nearby vectors, transmitting DENV to other close subjects which go to their homes and start a new peridomestic transmission cluster. On the other hand, young and elderly subjects, even though they probably develop greater force of infection, would have a lower participation in the local DENV dissemination because they have limited mobility, since younger individuals are typically symptomatics while older ones have lower mobility. In this scenario, the neighbors who live inside 50 meter-radio, although they have a lower risk of infection, are comparable in the dispersion of infection with subjects that live in the same house of the ICs, since they are much more than cohabitants. Therefore all the persons inside 50 meter-radio contribute in the dengue dispersion in these endemic communities.

This hypothesis provides an explanation to the relative ineffectiveness of perifocal measures; the opportunity of these measures is limited by the asymptomatic/symptomatic infections, underreported/undernotified cases and the spread of infection by asymptomatic or by a combination of those factors. Finally, the absence of case notifications from private care centers presents an opportunity for health services to improve the mexican dengue surveillance.

## Supporting Information

S1 Checklist STROBE Checklist. (DOC)

S1 Table Laboratory diagnostic of DENV recent infected subjects from de cohort study. (DOCX)

S2 Table Multilevel Pre-enrollment Infection House Model (n = 388 houses, 91 groups). (DOCX)
