# Supplementary material for: Peridomestic Infection as a Determining Factor of Dengue Transmission
Source: PLoS Negl Trop Dis. 2015 Dec 15;9(12):e0004296. doi: 10.1371/journal.pntd.0004296 (PMC4684393; doi:10.1371/journal.pntd.0004296)
Supplement: S1 Table — (DOCX) [file pntd.0004296.s002.docx]

**Supporting Information 1 Table. Laboratory diagnostic of DENV recent infected subjects from de cohort study.**

| **Infection** | **IgM capture** | **IgG capture** | **IgM e IgG capture** | **NS1**^a^ |
| --- | --- | --- | --- | --- |
| Pre-enrollment (n=213) | 66^a^ (31) | 93 (43.7) | 49 (23) | 5 (2.3) |
| Post- enrollment (n=40)^b^ | 12 (30) | 20 (50) | 5 (12.5) | 2 (5) |

^a^ One infection was diagnosed by seroconversion of IgG indirect ELISA Panbio.

^b^ 5 ELISA IgM test and all NS1 test were performed by SSM in subjects that present symptoms and were to the health service.
