# Supplementary material for: Peridomestic Infection as a Determining Factor of Dengue Transmission
Source: PLoS Negl Trop Dis. 2015 Dec 15;9(12):e0004296. doi: 10.1371/journal.pntd.0004296 (PMC4684393; doi:10.1371/journal.pntd.0004296)
Supplement: S2 Table — (DOCX) [file pntd.0004296.s003.docx]

**Supporting Information 2 Table. Multilevel Pre-enrollment Infection House Model (n=388 houses, 91 groups).**

| **Variable** | **Model 1 (with individual-level variables)**  **PRRa (95% CI)** | **Model 2 (with contextual-level variable)**  **PRRa (95% CI)** |
| --- | --- | --- |
| **Individual variables** |  |  |
| **Exposure to an IC** |  |  |
| Unexposed | 1 | 1 |
| Lives within 50m of an IC | **1.72 (1.22-2.44)** | **1.87 (1.33 - 2.62)** |
| Cohabits with an IC | **2.96 (1.99-4.41)** | **3.25 (2.20-4.81)** |
| **Location** |  |  |
| Tepalcingo | 1 | 1 |
| Axochiapan | **1.50 (1.07-2.11)** | **1.57 (1.14 – 2.17)** |
| **Toilet** |  |  |
| Direct discharge | 1 | 1 |
| Other | 1.23 (0.93-1.63) | 1.30 (0.99 – 1.71) |
| **Younger than 5 year old at home** |  |  |
| No | 1 | 1 |
| Yes | 1.30 (0.98-1.71) | **1.33 (1.01 – 1.74)** |
| **No. of cohabitants between 30 and 64 years old** | **0.83 (0.72-0.96)** | **0.83 (0.73 – 0.96)** |
| **Contextual variables** |  |  |
| **House Index (10 points %)** |  | **1.1 (1.03 – 1.17)** |
| **Log-likelihood value** | -329.60 | -153.03 |
| **AIC** | 675.21 | 670.13 |

**PRRa: Adjusted Prevalence Rate Ratio**
